# Optimizing inpatient bed management in a rural community-based hospital: a quality improvement initiative

**DOI:** 10.1186/s12913-023-10008-6

**Published:** 2023-09-18

**Authors:** Brian N. Bartlett, Nadine N. Vanhoudt, Hanyin Wang, Ashley A. Anderson, Danielle L. Juliar, Jennifer M. Bartelt, April D. Lanz, Pawan Bhandari, Gokhan Anil

**Affiliations:** 1 Regional Vice Chair of Clinical Practice, Mayo Clinic Health System – Southwest MN region, 1025 Marsh St., Mankato, MN 56002 USA; 2 Department of Finance, Mayo Clinic, Rochester, MN USA; 3 Chair, Hospital Internal Medicine, Mayo Clinic Health System – Southwest MN region, Mankato, USA; 4 Emergency Medicine, Mayo Clinic Health System – Southwest MN region, Mankato, MN USA; 5 Nursing Administration, Mayo Clinic Health System – Southwest MN region, Mankato, MN USA; 6 Hospital Operations, Mayo Clinic, Rochester, MN USA; 7 Vice Chair, Administration, Mayo Clinic Health System – Southwest MN region, Mankato, MN USA; 8 Department of Automotive and Manufacturing Engineering Technology, Minnesota State University, Mankato, MN USA; 9 Regional Chair of Clinical Practice, Mayo Clinic Health System – Southwest MN region, Mankato, MN USA

**Keywords:** Acute care beds, Capacity constraints, Community hospital, Inpatient bed management optimization, Patient transfers

## Abstract

**Background:**

Appropriate use of available inpatient beds is an ongoing challenge for US hospitals. Historical capacity goals of 80% to 85% may no longer serve the intended purpose of maximizing the resources of space, staff, and equipment. Numerous variables affect the input, throughput, and output of a hospital. Some of these variables include patient demand, regulatory requirements, coordination of patient flow between various systems, coordination of processes such as bed management and patient transfers, and the diversity of departments (both inpatient and outpatient) in an organization.

**Methods:**

Mayo Clinic Health System in the Southwest Minnesota region of the US, a community-based hospital system primarily serving patients in rural southwestern Minnesota and part of Iowa, consists of 2 postacute care and 3 critical access hospitals. Our inpatient bed usage rates had exceeded 85%, and patient transfers from the region to other hospitals in the state (including Mayo Clinic in Rochester, Minnesota) had increased. To address these quality gaps, we used a blend of Agile project management methodology, rapid Plan-Do-Study-Act cycles, and a proactive approach to patient placement in the medical-surgical units as a quality improvement initiative.

**Results:**

During 2 trial periods of the initiative, the main hub hospital (Mayo Clinic Health System hospital in Mankato) and other hospitals in the region increased inpatient bed usage while reducing total out-of-region transfers.

**Conclusion:**

Our novel approach to proactively managing bed capacity in the hospital allowed the region’s only tertiary medical center to increase capacity for more complex and acute cases by optimizing the use of historically underused partner hospital beds.

## Introduction

Establishing an ideal number of staffed inpatient beds is a complex issue for health care organizations. Numerous factors, such as planned and predicted inflows into the hospital system, contribute to this challenge. Historically, bed demand for elective and other surgical procedures requiring an inpatient bed has been easier to predict than that for admissions from the emergency department, urgent care, and clinic visits [1]. Other variables, such as labor markets, space constraints, and equipment use, add to the complexity of predicting bed demand. Inadequate inpatient bed availability has led to emergency department crowding and, in turn, concern for the quality of services delivered [2, 3]. These factors combine to create a challenging operational workflow when managing inpatient bed usage [4, 5].

Our hypothesis centered on the strategic allocation of beds among various hospital sites. Specifically, we hypothesized that proactive bed assignment at tertiary centers for patients with presenting signs of severe illness who require specialized care unavailable at smaller sites will enhance regional health care provision. Concurrently, we hypothesized that optimizing the capacity at smaller hospitals by admitting patients with non-severe illness will improve patient distribution and thereby augment the ability of hospital systems to deliver comprehensive care throughout a health care region. To test this hypothesis, we developed a novel approach (via a combination of various methods) to bed hold to ensure that hospital beds were available for patients with severe illness (those requiring specialized services such as Cardiology, Neurosurgery, Orthopedic Surgery, Trauma Care, Interventional Radiology or Gastroenterology procedures) at the Mayo Clinic Health System (MCHS) hospital in Mankato, Minnesota, USA (MCHS-Mankato). This dual-pronged approach anticipates both unplanned, acute admission requirements and the ongoing need for beds for less critical cases. MCHS-Mankato is the only hospital that provides acute care, primary care, continuous emergency care, a level II nursery, critical care, advanced trauma care, and specialized medicine in southwestern Minnesota. Therefore, reducing or preventing transfers to outside of the region and providing care to patients at the right place and at the right time were important aims of our study. However, certain emergency situations required transfer to another tertiary hospital in which advanced care is provided.

The Admission Transfer Center (ATC) at MCHS acts as a critical node for patient distribution, operating like an air traffic control center to manage patient placement. The ATC, staffed by triage nurses at a centralized Southern Minnesota location, connects to all MCHS sites via an advanced information technology network. It uses data-driven strategies to facilitate timely transfers and improve patient flow, which contributes to operational efficiency. Additionally, its unique model provides a rich data source for investigating the dynamics of patient transfers, which thereby fosters various research opportunities.

## Methods

### Setting

The MCHS Southwest Minnesota region (SWMN) is based on a hub-and-spoke model that includes 2 acute care hospitals and 3 critical access hospitals. MCHS-Mankato is the only tertiary medical center and is the *hub* in this region. Four *spoke* hospitals in the region (Fairmont, St James, New Prague, and Waseca, Minnesota) serve patients with less severe illness. MCHS-Mankato consistently had greater than 95% occupancy and remained in diversion status on most days since the COVID-19 pandemic began because of a combination of increased demand, bed closures, staffing shortages, and other challenges. During the same period, tertiary hospitals throughout Minnesota also had high occupancy rates, which limited their capacities to accept patient transfers. These limitations resulted in previously unseen delays in hospital care throughout southwest Minnesota.

In SWMN, patients requiring care for severe illness and subspecialty care could often be served only at MCHS-Mankato. Therefore, these patients would be transferred out of the region when no inpatient beds were available at MCHS-Mankato. At the start of this project, the spoke hospitals in SWMN were not consistently reaching full occupancy. The historical method of admitting patients to MCHS-Mankato until full capacity was reached failed to make use of open spoke hospital beds for appropriate patients.

### Study process

In the third quarter of 2021, MCHS-Mankato started the design and implementation of a project to optimize hospital bed management in SWMN. This project aimed to ensure the availability of beds for local patients with severe illness, maximize capacity opportunities in the surrounding spoke hospitals, and reduce out-of-region transfers. The spoke hospitals, all under the purview of MCHS leadership and operationally integrated via the ATC, were pivotal as key stakeholders in the formulation of our study design.

**Fig. 1:**
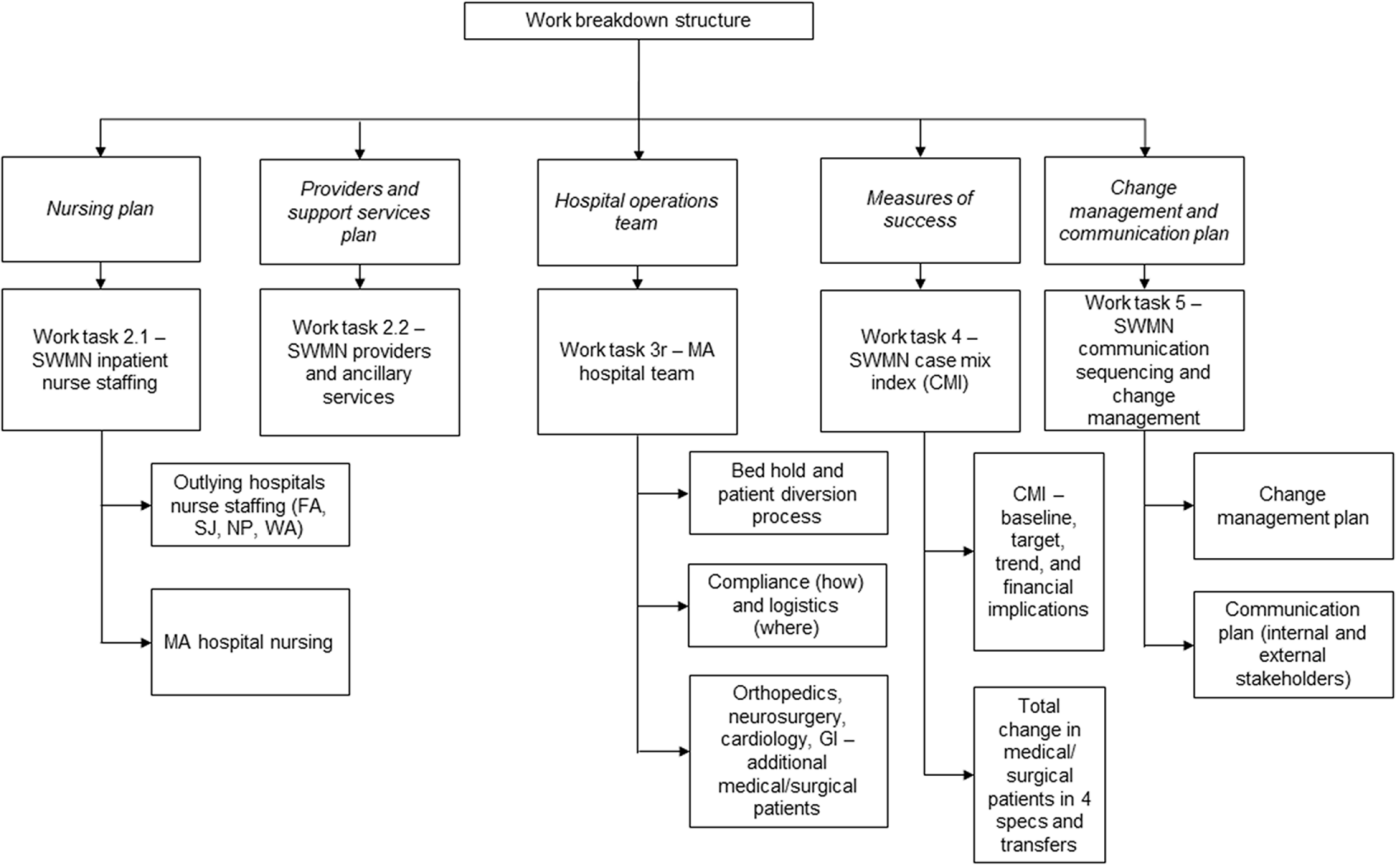
Work Breakdown Structure. CMI indicates case mix index; FA, Fairmont, Minnesota; GI, gastrointestinal; MA, Mankato, Minnesota; NP, New Prague, Minnesota; SJ, St James, Minnesota; SWMN, Mayo Clinic Health System – Southwest Minnesota region; WA, Waseca, Minnesota

The initiative for optimizing hospital bed management was divided into 2 phases: phase 1, which comprised discovery (opportunity identification), planning, and proposal steps, and phase 2, which consisted of implementation. Through direct engagement with key stakeholders, phase 1 of the project followed the project engagement approach outlined in Table 1. This approach was closely aligned with Agile methodology and principles and our work breakdown structure (Fig. 1) to yield our overarching deliverables in the form of tasks or work buckets. In phase 2 of the project, 4 work streams were proposed after brainstorming sessions with subject matter experts and key stakeholders. The 4 work streams included the pre-admission process, transfer plan, updated admission guidelines, and updated diversion plan (Table 2). These work streams were led by respective operations, nursing, and executive partners. Each work stream was critical to ensuring that all aspects of hospital input were addressed in pursuit of optimizing bed management.

**Table 1:**
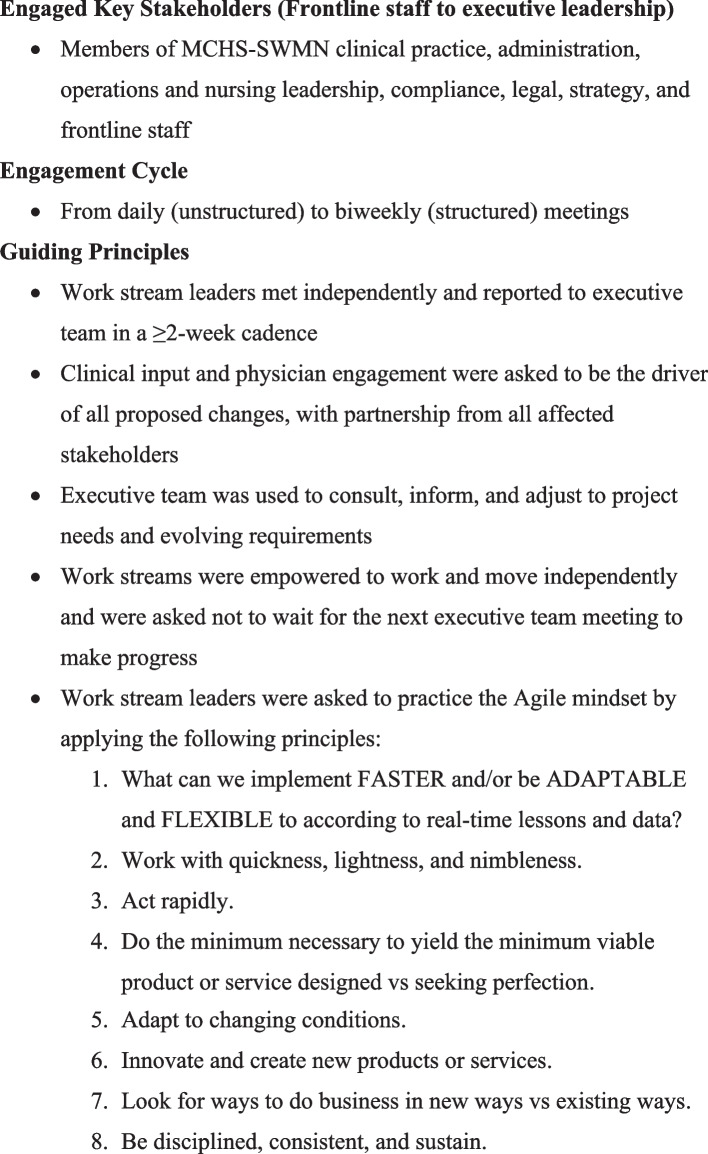
Project Engagement approach

*Abbreviation*: *MCHS-SWMN* Mayo Clinic Health System – Southwest Minnesota region

**Table 2:** Optimization of bed management project work streams

Work stream deliverable	Timeline	Tactics
Pre-admission process^a^	January 1 through March 31, 2022	Progressive care unit Pre-admission process for surgical patients before 12 pm
Transfer plan^b^	January 1 through June 30, 2022	ED lateral transfer process under way Scripting on capacity and capability for staff MATC partnership to triage patients to regional sites EMS assessment
Updated admission guidelines^c^	January 1 through June 30, 2022	Criteria outlined to reserve beds for patients with severe illness in 4 strategic service lines
Updated diversion plan	January 1 through June 30, 2022	Diversion service area Diversion activation Hospital bed management optimization dashboard

*Abbreviations*: *ED* Emergency department, *EMS* Emergency medical services, *MATC* Midwest Admission and Transfer Center, *PCU* Progressive care unit

^a^Pre-admission for surgical procedures planned the night before admission

^b^Redirection of patients from sites other than Mayo Clinic to regional sites and from the Mayo Clinic Health System hospital in Mankato, Minnesota, to regional sites in the Southwest Minnesota region

^c^Level of care and specialty beds

### Deliverables and procedures

Deliverables were designated for each of the 4 work streams, with a project leader and key stakeholders assigned from various work areas to assess the current operational state and identify opportunities for improvement.

The pre-admission process work stream was identified as an essential component because it is a source of planned admissions, with an aspect of volume control structured by the hospital. The pre-admission process includes surgical and procedural patients requiring hospital care. The transfer plan work stream investigated the systems and processes used by MCHS-Mankato to leverage its partnership with the internal patient transfer center, external hospital transfer centers, and the emergency medical system to direct and triage patients awaiting admission into hospitals that have the capability and available bed capacity to admit patients.

The admission guidelines work stream was heavily focused on the admission criteria that MCHS-Mankato and the spoke hospitals have established to admit patients according to their respective capabilities. This exercise provided an opportunity to distinguish between patients with various levels of illness severity and quantify the volume or demand for inpatient care. These criteria were used to prioritize a selection of beds to ensure safe and timely placement of patients with critical illness. The diversion plan work stream leveraged what we learned from each of the previous deliverables to identify a diversion service area and activation/deactivation criteria for hospital and/or service line diversions. Each work stream deliverable had an essential component for structuring hospital bed optimization, with interdependencies between each component.

#### Pre-admission process

Throughout the COVID-19 pandemic, access to critical care beds was considerably reduced because of unprecedented demand. As surgical case volume began to quickly increase, implementing a process that allowed local teams to adequately plan for surgical admissions that require postoperative critical care placement became essential. Historically, the routine ebb and flow of hospital discharges provided adequate capacity for surgical admissions. However, the substantial effect of the COVID-19 pandemic on capacity necessitated a change in practice. To support the timely placement of postoperative critical care patients, a workflow was established that provided advanced notification of all planned cases by the level of care to patient placement teams. The night before a surgical procedure, a systems-based software program generated a bed request that was visible to the bed planning team for all scheduled cases. As part of the enhanced pre-admission process, bed planning staff pre-assigned critical care surgical patients to an inpatient bed for cases completed before 4 pm. This was a fluid process, allowing for analysis of emergent situations with an immediate need for a critical care bed in the hospital. Teams would reevaluate the pre-admitted bed to balance the emergent need and anticipated surgical patient. Pre-assigned patients were factored into hospital occupancy, which was therefore directly associated with diversion status. This practice allowed SWMN hospitals to mitigate capacity constraints through advanced planning.

#### Transfer plan

A vital patient-based perspective was incorporated into our research design through the engagement of both our MCHS-Mankato and SWMN regional boards. These entities not only granted approval for our initiative but also actively encouraged its execution, which effectively served as a patient advisory board. Furthermore, our Emergency Medical Treatment and Labor Act compliance team conducted a thorough review of the transfer policy. The team’s endorsement stemmed from the optimized regional capacity to provide an advanced triaging system, which thereby ensured that the most critically ill patients were less likely to require transfer out of the SWMN region.

SWMN established a plan to determine the best location for a patient to be admitted to an inpatient setting throughout the region according to clinical needs and capacity. Because of the development of regional admission guidelines, physicians and practitioners understood the capabilities of all spoke hospitals. Each of the critical access spoke hospitals also reinforced staffing plans to accommodate patients who did not require a high level of care, which permitted admissions during capacity restraints. Emergency department physicians and practitioners were made aware of bed capacity for the day through secure chat messaging. This allowed practitioners to identify the level of care a patient required and to determine which location could best meet the needs of the patient. If a current location had no capacity or ability to treat the patient, the patient was then parallel transferred to another location in our region for inpatient admission. This transfer plan improved care for patients by allowing them to remain in the region for treatment while also maximizing the capacity of all hospitals in the region.

#### Updated admission guidelines

SWMN admission guidelines were established to support safe patient placement according to site, unit, and nurse capability, which maximized bed usage for each hospital in the region. The guidelines delineated the placement of patients on the basis of their specific clinical needs and identified which units could provide care, as well as those that could not provide care. The guidelines were analyzed and benchmarked against other hospital facilities for best practices.

Admission guidelines served as the foundation for optimizing hospital bed management, which established a precedent for ensuring that each patient was placed in the right bed after admission. Historically, patients without severe illness may have been placed in beds designated for patients with severe illness when no other internal capacity options were available. This practice reduced critical care bed capacity, contributed to the underuse of regional beds, and negatively affected hospital patient flow. Work stream reevaluation of admission guidelines indicated that restricting the critical care units for patients with severe illness who met the identified admission criteria was a foundational component to optimizing SWMN hospital bed management.

#### Updated diversion plan

The diversion plan was the most complex work stream. Admission patterns throughout SWMN were studied. An analysis of admission guidelines and patient flow patterns throughout each of the 5 SWMN hospitals identified that underuse of beds at regional spoke hospitals occurred in conjunction with overcapacity at MCHS-Mankato. Patients requiring high-level and specialty care had barriers to bed access at MCHS-Mankato because of a lack of capacity. Specific specialty practices (ie, service lines), including orthopedics, cardiology, neurosurgery, and gastroenterology, were disproportionately affected by these capacity constraints. Because MCHS-Mankato is the only multispecialty tertiary care center in an approximately 80-mile radius, patients unable to access inpatient care were transferred distantly outside of the SWMN region. This placed a considerable strain on local emergency medical service resources, which resulted in a systemwide patient flow deficit. Admission pattern analysis indicated that an average of 23 patients per day without severe illness who would have met the admission criteria of regional spoke hospitals occupied a bed in MCHS-Mankato. These beds were identified as potential capacity for patients with severe illness at MCHS-Mankato.

To maximize spoke hospital bed usage, it was essential to shift from the traditional approach of admitting patients to MCHS-Mankato on a first-come, first-served basis until full capacity was reached. The foundational practices implemented by the other 3 work streams readied the teams for the next step (ie, enhancing the diversion procedure) in optimizing hospital capacity throughout the region. To reduce out-of-region transfers of patients with serious illness, specific thresholds were established that activated diversion procedures according to each level of care, including intensive, progressive, and medical/surgical care. When a diversion procedure was activated, specific criteria were used to prioritize admission for select service area hospitals, levels of care, and tertiary services to maximize use of the limited volume of beds. This action preserved local bed capacity for more critical care patients, which improved quality, reduced the distance of transfers, and minimized the overall strain on the regional health care system.

Diversion thresholds were established after an in-depth analysis of patient demand, which included historical admission, discharge, and transfer rates for each level of care and primary admission services. Service line diversion was activated when the facilitywide medical-surgical bed volume was reduced to 4 beds. These beds were then reserved for patients in the diversion service area who required inpatient care from orthopedics, neurosurgery, cardiology, or gastroenterology because these service lines had the highest frequency of out-of-region transfers. When a request for admission was made, bed management would perform a real-time bed capacity assessment and connect the hospital medicine physician or admitting specialist with the referring clinician for admission screening. If the patient did not require care through the service lines, transfer options were pursued to maximize regional bed capacity. Occasionally, exceptions were made at the discretion of the hospitalist and emergency department physician. Diversion thresholds for high-level care areas were activated when bed volume was reduced to 3 beds in progressive care and to 2 beds in intensive care. Admission to either progressive or intensive care was limited to the regional service area during the diversion but not to specific service lines.

#### Implementation plan summary

The project was implemented with a series of Plan-Do-Study-Act (PDSA) cycles in 2 trials performed during the second quarter of 2022. Trial 1 was conducted from April 27, 2022, through May 3, 2022, in which the new diversion procedure was implemented. Trial 2 was performed from June 1, 2022, through June 7, 2022. Because of the success of trial 1, we continued use of the new diversion procedure during the period between trials 1 and 2. A key change from trial 1 to trial 2 was allowing a thorough review of exceptions to the established admission criteria during diversion activation. Patients requiring interventional radiology or expert trauma care were not excluded in trial 2 but were excluded in trial 1. The hospitalist team was allowed to make 2 exceptions per day to the admission criteria, when appropriate.

### Project outcomes and analysis

Measures of success were identified in 2 primary categories (transfer and census). Transfer rates were calculated by dividing transfer volume by all admissions (transfer volume plus total admissions) of all adult patients (excluding behavioral health patients) during the period. Transfer rates were determined for total transfers from all SWMN hospitals (MCHS-Mankato alone and the 4 spoke hospitals combined); transfers from SWMN hospitals (MCHS-Mankato alone and the 4 spoke hospitals combined) to Mayo Clinic in Rochester, Minnesota; and parallel transfers from MCHS-Mankato to SWMN spoke hospitals or to hospitals in the MCHS Southeast Minnesota region (SEMN). Transfer rates after each implementation trial/PDSA cycle were compared with the average 2021 transfer rate (ie, baseline).

## Results

### Trial 1 outcomes

**Fig. 2:**
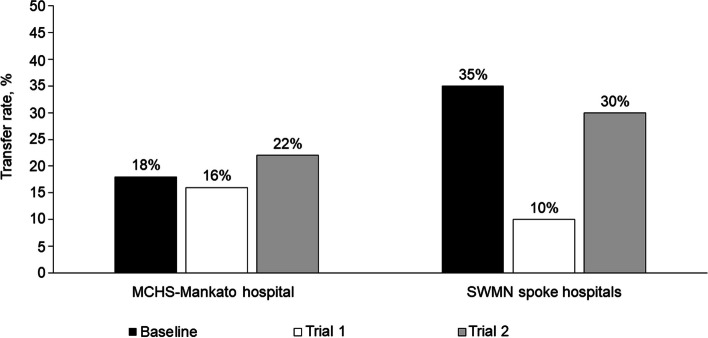
Transfer Rates for Mayo Clinic Health System (MCHS) Southwest Minnesota (SWMN) Regional Hospitals. Adult patients (excluding behavioral health patients) were transferred from the MCHS hospital in Mankato, Minnesota, or 1 of the 4 SWMN spoke hospitals to an external hospital, a hospital in another MCHS region, or Mayo Clinic in Rochester, Minnesota. Total transfer rates for all causes were determined for the 2021 calendar year (baseline) and during 2 project trial periods of 7 days

**Fig. 3:**
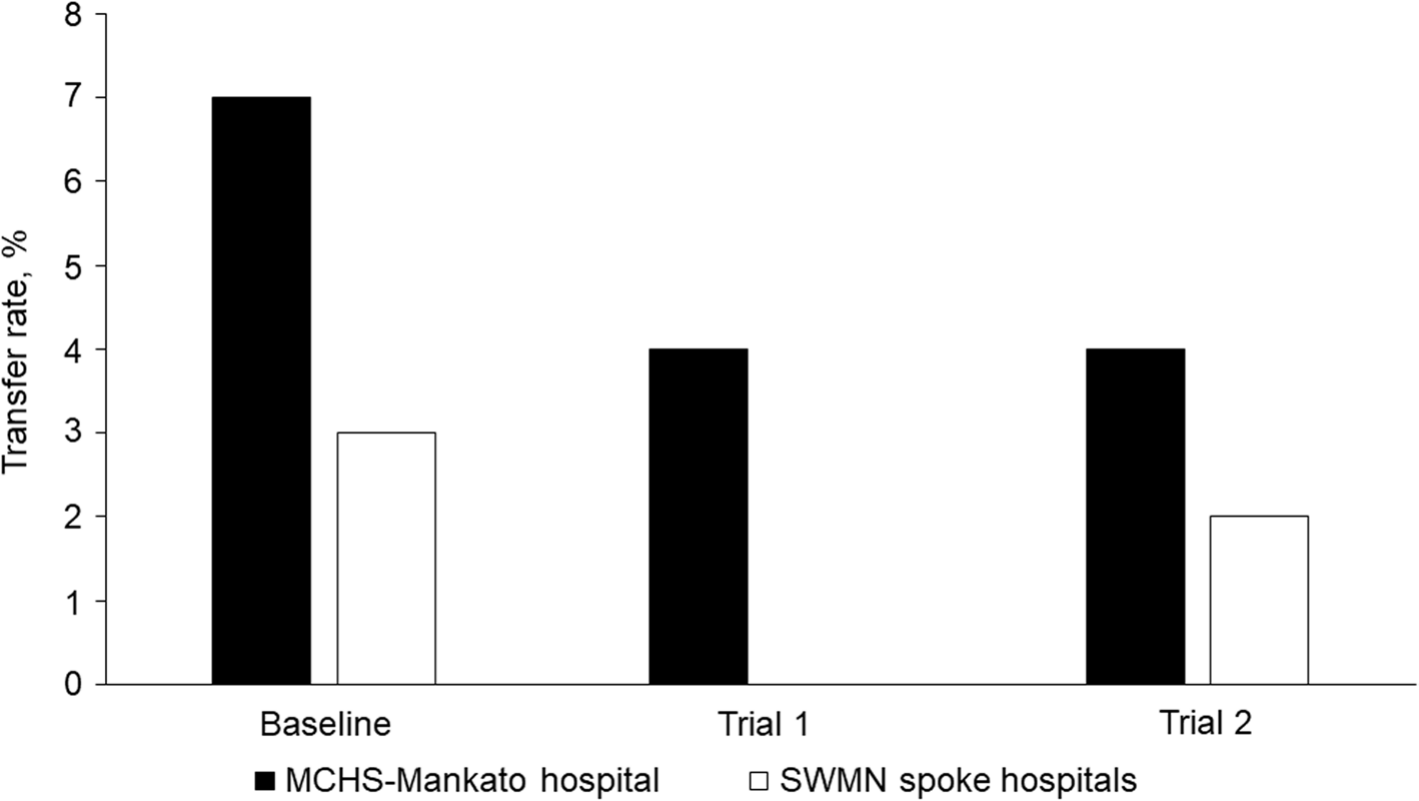
Transfer Rates of Patients Transferred to Mayo Clinic in Rochester, Minnesota. Adult patients with acute illness (excluding behavioral health patients) were transferred from the Mayo Clinic Health System (MCHS) hospital in Mankato, Minnesota, or 1 of the 4 MCHS Southwest Minnesota spoke hospitals to Mayo Clinic in Rochester, Minnesota. Transfer rates were determined for the 2021 calendar year (baseline) and during 2 project trial periods of 7 days

**Fig. 4:**
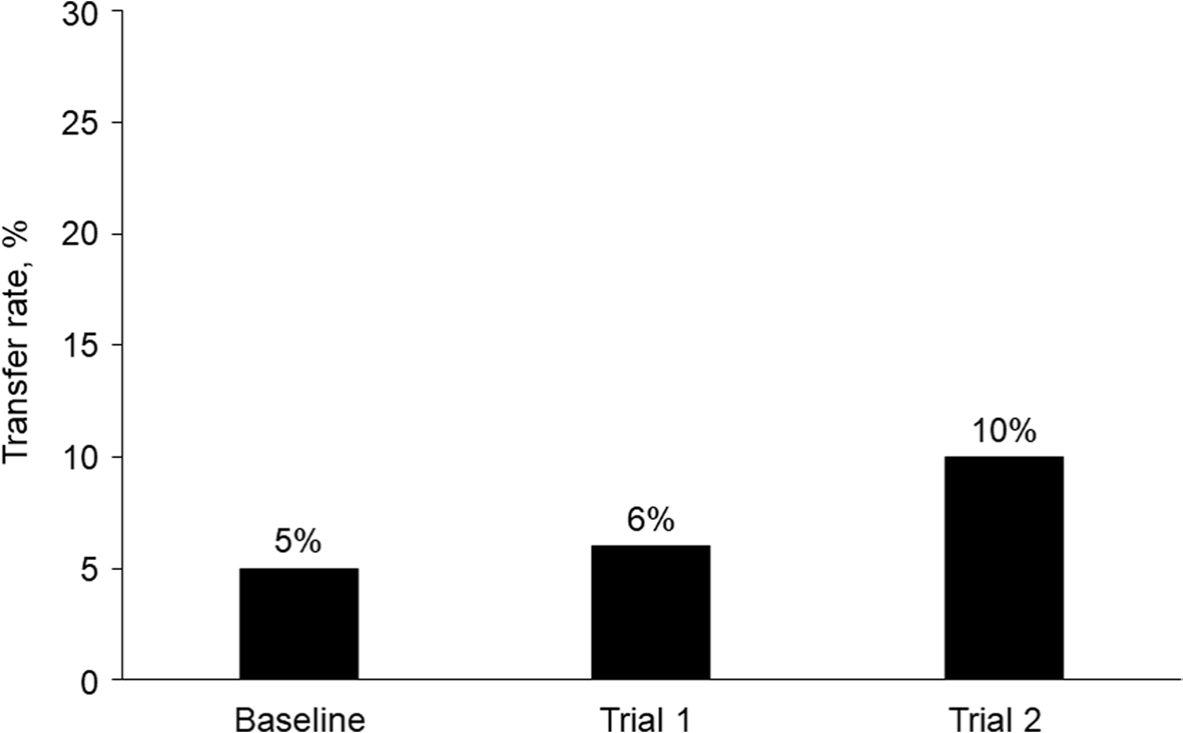
Parallel Transfer Rates Among Mayo Clinic Health System (MCHS) Hospitals. Adult patients (excluding behavioral health patients) were transferred from the MCHS hospital in Mankato, Minnesota, to hospitals in the MCHS Southwest Minnesota (SWMN) and Southeast Minnesota regions. Parallel transfer rates were determined for the 2021 calendar year (baseline) and during 2 project trial periods of 7 days

During implementation trial 1, the reduction from baseline in the total, all-cause transfer rate of patients out of the region from the 4 spoke hospitals (3 critical access hospitals and MCHS-Fairmont) was 71%, which changed from 35% at baseline to only 10% after trial 1 (Fig. 2) In addition, the average daily census of patients with severe illness who were admitted through the prioritized service lines (orthopedics, neurosurgery, cardiology, and gastroenterology) to MCHS-Mankato remained the same (*n* = 37) during both trial 1 and trial 2, despite the reduced number of available beds due to bed blocking. The rate of transfers from MCHS-Mankato to Mayo Clinic in Rochester, Minnesota, a destination medical center, decreased by 43%, from 7 to 4% (Fig. 3). Additionally, the rate of parallel transfers from MCHS-Mankato to SWMN and SEMN increased by 20% from a baseline of 5% to 6% (Fig. 4).

### Trial 2 outcomes

Considerable inpatient nurse staffing shortages occurred during trial 2, which did not occur during trial 1. This resulted in closure of an average of 22 inpatient beds per day (20% reduction) during trial 2, which further reduced hospital capacity. However, MCHS-Mankato continued to admit 97% of our average historical admission volume. During trial 2, the rate of all-cause transfers from MCHS-Mankato increased by 22% from baseline (Fig. 2), but the mean transfer rate of patients from MCHS-Mankato to Mayo Clinic in Rochester, Minnesota, decreased from a baseline of 7% to 4% (Fig. 3). Although service line admission volume did not increase during trial 2, the mean daily census for all 4 service lines increased from 0 beds at baseline to 30 beds after trial 2, despite inpatient capacity reduction. Parallel transfers from MCHS-Mankato to the SWMN spoke hospitals and SEMN hospitals increased from 5% at baseline to 10% during trial 2 (Fig. 4).

## Discussion

The complexity of treating patients requiring hospital care has been increasing, while the challenges of providing continuous staffing in hospitals have been mounting for health care organizations. Many recommendations on how hospitals can improve their performance despite these challenges have been proposed [6]. Improvements in patient flow and movement pathways, as well as care coordination through optimization of admission and discharge processes, are methods that can positively affect quality and patient care outcomes [7]. Intentionally delineating roles among hospital frontline staff and management teams has also shown positive results [8]. Our study used a novel strategy of combining some of these well-documented recommendations with new strategies to optimize our regional inpatient service, which routinely exceeds 85% capacity.

The PDSA cycle outcomes of the 2 implementation trials yielded results that supported our hypothesis. These findings suggest that hospital systems may benefit from proactively assigning beds for patients with severe illness at their tertiary centers and optimizing capacity at their smaller hospital sites by admitting patients without severe illness. Often, hospital systems will fill available capacity on a first-come, first-served basis. This approach prioritizes speed of admission and discounts matching patient needs with medical and surgical resource availability at the right location and time.

Research regarding the strategy of proactively transferring patients between acute care hospitals and non-acute care hospitals in a health system appears to be limited. However, some studies have reported that patients with complex conditions and who have been transferred to another hospital may have poor outcomes [9, 10]. Traditionally, patient transfers occur only after a tertiary hospital activates diversion procedures. Our findings suggest that identifying bed demand for patients with severe illness should not be limited to intensive and progressive care units only. Alternatively, a clearly delineated projection of bed demand for patients who require complex services must be considered when creating operational capacity designations.

Although our preliminary findings indicated a positive outcome of our quality improvement initiative, these findings were limited by an increase in bed shortages because of staffing constraints and fluctuating patient volumes in clinics, urgent care centers, and emergency departments. The bed shortages that occurred during the second implementation trial could have affected the integrity of our optimization project data. Increased barriers to inpatient bed access also challenged the enhancement of the exception criteria process, which remains an opportunity for improvement. Emergency medicine practitioners felt particularly challenged in finding recipient hospitals for patients in a timely manner. However, MCHS leadership helped reinforce the value of this project, which prioritized bed access for patients with severe illness and thereby reduced the risk of boarding or transferring critical care patients. Our analysis also lacked sufficient statistical power to establish significant differences. Despite these limitations, our results suggest that our project to optimize hospital bed management in a regional health care system had a generally positive effect.

Operational inefficiencies in the ATC and transfer delays posed some challenges. Future ATC flow enhancements and more robustly staffed ambulance services may help mitigate these issues. Our spoke hospitals were crucial for developing this process and were engaged partners in the trials. The primary challenge was ensuring timely patient transfers from the emergency department and managing the ATC operational flow. Envisaging further collaboration between the ATC and emergency medical service, we aim to use their expertise for process streamlining, enhancing the patient experience, and creating a more coordinated system in SWMN.

Future iterations of regional capacity optimization studies will most likely include analytical forecasts, such as inpatient service line demand beyond the current projection of 6 to 8 weeks [11–17]. Bed designations can remain fluid and responsive to analytical projections for months and even years and as hospital data analytics develop. A better understanding of population health needs and advanced at-home care options, including advanced digital health and telemedicine offerings, will affect future hospitalization needs and forecasting of inpatient bed usage.

## Conclusion

Some hospital systems may benefit from proactively transferring patients to hospitals in their system that primarily serve patients without severe illness at an early time in the admission cycle and reserving beds at a larger tertiary medical center for patients with severe illness. The traditional approach of filling a hospital, activating diversion procedures, and then seeking alternative placement fails to maximize resource capabilities in a large regional system. As capacity constraints continue to strain hospital systems, innovative approaches to the admission process must be explored. Historical operational models that are relevant to only a single hospital fail to meet the demands resulting from contemporary resource constraints.

## Data Availability

The data supporting the conclusions of this article are included within the article.

## Abbreviations

ATC: Admission Transfer Center
MCHS: Mayo Clinic Health System
MCHS-Mankato: Mayo Clinic Health System hospital in Mankato, Minnesota, USA
PDSA: Plan-Do-Study-Act
SEMN: MCHS Southeast Minnesota region
SWMN: MCHS Southwest Minnesota region
